# Oral pathologies and underweight conditions among people living with HIV/AIDS in a health facility in Yaoundé, Cameroon: a study of 205 cases

**DOI:** 10.1186/s40795-024-00835-z

**Published:** 2024-02-15

**Authors:** Lionel Berthold Keubou Boukeng, Léonie Dapi Nzefa, Djouwairiyatou Sali, Claude Axel Minkandi, Jean Yves Bevela, Ariane Nouko, Charly Etoa Ebogo

**Affiliations:** 1 Department of Public Health, Faculty of Medicine and Biomedical Sciences, University of Yaoundé I, Yaoundé, P.O. Box 1364, Cameroon; 2 Department of Medicine and Optometry, Inst of Health and Life Sciences, Linnaeus University, Kalmar, P.O. Box 39182, Sweden

**Keywords:** Cameroon, Oral pathologies, Underweight, HIV

## Abstract

**Background:**

Human immunodeficiency virus (HIV) infection is a public health concern worldwide. The clinical manifestations include underweight and oral lesions. The objective of this study was to assess the relationship between oral pathologies and underweight among HIV-positive patients in Yaoundé, Cameroon.

**Methods:**

We conducted a cross-sectional study between February 1st and 30th June 2021 at Yaoundé Central Hospital in Cameroon. A total of 205 HIV positive patients aged at least 18 years were recruited via consecutive sampling. The questionnaire consisted of sociodemographic information, anthropometric data, dietary habits, HIV history and treatment and oral examination data. The data were analysed with R software. Multivariate analysis was used to assess the risk of being underweight among HIV-positive patients with oral pathologies. A *p* value < 0.05 was considered to indicate statistical significance.

**Results:**

The prevalence of oral pathologies was 52.7% (95% CI: 45.6–59.6), and the main pathologies were candidiasis (40.5%, 95% CI: 33.7–47.5) and linear erythema (32.2%, 95% CI: 25.9–39.1). The prevalence of underweight was 20% (95% CI: 14.88–26.26). Binary logistic regression revealed that HIV-positive patients with oral pathologies were 10.89 (95% CI: 2.28–16.63) times more likely to be underweight than were HIV positive and AIDS patients without oral pathologies (*p* = 0.002).

**Conclusion:**

Oral candidiasis and linear erythema were common in HIV positive and AIDS patients. HIV-positive and AIDS patients with these oral pathologies were at higher risk of being underweight than were those without oral pathologies. The effective medical care of these patients must include oral and nutritional management.

**Supplementary Information:**

The online version contains supplementary material available at 10.1186/s40795-024-00835-z.

## Background

Human immunodeficiency virus (HIV) infection is a global public health concern [1]. According to the United Nations AIDS Program (UNAIDS), the number of HIV-positive people worldwide in 2021 was estimated to be 38.4 million [1]. West and Central Africa are the third most affected regions, with 45 million people infected [1]. In Cameroon, the HIV incidence was estimated to be 2.7% among adults aged 15 to 49 in 2018 [2]. Spectrum projections in 2020 revealed that the number of HIV-positive people was 504,472 [2].

Two classifications are used to describe the course of the infection: those of the Centers for Disease Control and Prevention (CDC) and the World Health Organization (WHO) [3, 4]. Clinical manifestations include being underweight, described as weight loss of less than 10% at WHO stage 2, then greater than 10% at stage 3 and cachectic syndrome during the fourth stage [5]. Several factors, including poor immunological status, lack of compliance with antiretroviral treatment and the presence of oral pathologies, have been associated with this progressive weight loss [5, 6]. HIV attacks the immune system, leading to destruction of the host’s defence cells, and in its advanced phase, acquired immune deficiency syndrome (AIDS) occurs [5, 6]. This immunosuppression leads to the occurrence of opportunistic infections, including oral pathologies [5]. Oral pathologies may be present in more than half of infected individuals and more than 80% of patients in the AIDS phase [5–7]. Oral pathologies may be fungal, viral, bacterial, or tumorous may take the form of nonspecific ulceration [5, 8]. Seven cardinal lesions, namely, candidiasis, hairy leukoplakia, Kaposi’s sarcoma, linear gingival erythema, ulcerative necrotizing gingivitis, ulcerative necrotizing periodontitis and non-Hodgkin’s lymphoma, have been identified as strongly associated with HIV infection [7]. The clinical presentations of these pathologies depend on their location in the oral cavity, and the most recurrent symptoms are discomfort, pain, burning, dysphagia and difficulty eating properly [6]. This could result in a change in their diet, which can lead to a reduction in weight status in the long-term.

To our knowledge, little information is available on the relationship between oral pathologies and underweight in HIV-positive patients. Early detection and management of oral pathologies can improve HIV-positive patients’ quality of life. According to the WHO, oral pathologies are among the first clinical manifestations of the disease, and weight status is one of the best indicators for monitoring HIV infection [6]. The objective of this study was to assess the relationship between oral pathologies and underweight in HIV-positive patients at Yaoundé Central Hospital (YCH) in Cameroon.

## Methods

### Study design

We conducted a cross-sectional study.

### Study period and setting

The study took place at the day hospital department of YCH. The day hospital department is a specialized treatment center dedicated to monitoring HIV-positive patients in the city of Yaoundé. The study took place between February 1st and June 30^th,^ 2021.

### Study population

Our study population included all HIV positive patients attending the YCH. All HIV positive and AIDS patients aged at least 18 years who provided informed consent were included. Recruitment was consecutive. The sample size was obtained using the following formula:

$$ N={\left(\frac{\text{z}1-{\alpha } /2 \sqrt{\text{P}\left(1-\text{P}\right)}}{\text{d}}\right)}^{2},$$ $$

with the absciss of the normal distribution curve corresponding to the 95% confidence level of 1.96, 5% as the margin of error, a precision level of 5% and a prevalence of oral lesions in HIV positive people of 87.5%, as reported in Mali in 2017 [7]. The size was adjusted to 20% to take into account withdrawals from the study. A sample size of 205 was obtained.

### Data collection

The data were collected using a questionnaire developed for this study (see supplementary file). It was divided in five sections.

#### Sociodemographic characteristics

The first section included sociodemographic characteristics such as age reported in years, gender (male and female), occupation (primary, secondary and University) and level of education (employed, student and unemployed).

#### Clinical data

This second section was related to the HIV history and we recorded the date of initiation of treatment, the treatment regimen according to HIV management national guidelines which recommend three lines: first (Tenofovir,TDF/Lamivudine,3TC/Dolutegravir,DTG), second (Abacavir,ABC ou Zidovudine, AZT/Lamivudine, 3TC + Atazanavir boosted with Ritonavir,ATV/r ou Lopinavir boosted with Ritonavir, LPV/r) and third (Darunavir boosted with Ritonavir, DRV/r) + Dolutegravir (DTG) ± 1–2 nucleotide reverse transcriptase inhibitors) [8]. Then, we recorded the CD4 T-cell count (cells/mm^3^) of patients less than six months old to assess the patients’ immunological status (< 200, 200 to 500, ≥ 500) and clinical stage (stage 1 to 4) according to the World Health Organization (WHO) [4].

#### Oral examination procedure

The third section was used for the oral examination, which was carried out using a mouth mirror, a tongue depressor, a N°17 dental probe, a WHO periodontal probe graduated from 3.5 to 5.5 mm with a 0.5 mm diameter ball tip, and a forehead lamp [9]. The decayed missed tooth filled tooth (DMF) index was evaluated by adding the number of decayed teeth to the number of absent and filled teeth. Oral hygiene was assessed using the plaque index. The amount of dental plaque on the dental crown and marginal gingiva was assessed by classifying the plaque into four levels: no plaque, thin layer, visible plaque deposit and plaque accumulation [6, 7]. Oral lesions were identified, and gingival and periodontal pathologies were classified using the 2018 American Academy and European Federation of Periodontology criteria [9].

#### Anthropometric measurements

In this section, measurements were taken by qualified nurses according to WHO standard methods [10–12]. Participants’ weight was measured in kilograms to the nearest 0.1 kg using a standard scale calibrated to zero before and after each measurement [13, 14]. Height was measured in cm to the nearest 0.1 cm using a stadiometer; participants were asked to remove their shoes, stand up straight and look straight ahead in the Francfort plane [15–17]. Body mass index (BMI) was used as an indicator of nutritional status by dividing body weight (kg) by the square of height (m^2^). The BMI was classified into four categories according to the WHO: obese (BMI ≥ 30), overweight (25 < BMI ≤ 29.9), normal (18.5 < BMI ≤ 24.9), and underweight (BMI ≤ 18.5), with mild (17 < BMI ≤ 18.4), moderate (16 < BMI ≤ 16.9) and severe (BMI < 16) [11, 18, 19].

#### Nutritional status measurements

In the fifth section, dietary habits were collected using the 24-h recall method. This format searched for all foods and beverages consumed in the previous 24 h over a seven-day period [20].. The food consumption score (FCS), an index developed by the World Food Program (WFP), which provides a picture of a given period of the usual diet of an individual or a community, was subsequently calculated [21, 22]; this score lists the foods consumed over the last seven days and weights them according to their relative nutritional value. In this estimate, caloric intake is captured by the frequency of consumption and nutritional quality by the diversity of food groups [21, 22]; all frequency values above seven have been capped at seven. Foods were grouped into eight groups: cereals /starches, pulses, vegetables, fruits, meats/fish, milk, oil, and sugar/fatty foods. The sum of the weighted scores gives the overall score [21, 22]. Our participants were classified as having a poor diet (inadequate in quantity and quality) if the value was ≤ 21, those with a score between 21 and 35 were considered to have a limited diet and inadequate only in quality, and those with an FCS > 35 were considered to have an acceptable diet (adequate in quantity and quality) [21, 22].

### Ethical considerations

Ethical clearance and permission were obtained from the Ethics Committee of the Faculty of Medicine and Biomedical Sciences of the University of Yaoundé 1, Cameroon, with authorization number N°154/UY1/FMSB/VDRC/DAASR/CSD. The administrative authorization N°204/21/AR/MINSANTE/SG/DHCY/CM/SM was issued by the director of the Yaoundé Central Hospital. Written informed consent was obtained from all participants beforehand, and this study was performed in accordance with the Helsinki Declaration. Verbal assent was obtained from those between 18 and 20 years, and written consent was obtained from their parents. All the information collected from the participants was kept confidential, with no trace of identification details in the final report. HIV positive patients with oral pathologies were informed about their oral health status and offered a treatment plan.

### Statistical analysis

The data collected were analysed using CSPro 7.5 and analysed using R version 4.3.1. Qualitative variables are presented as frequencies and percentages. Quantitative variables with a normal distribution are summarized with their means. Bivariate analysis was performed with underweight as the dependent variable; the presence of oral pathologies, sociodemographic information, clinical and immunological characteristics and the food consumption score as the independent variables. Variables with a significant *p* value or close significance were subsequently included in the binary logistic regression model. The stepwise approach was used, and the model with the lowest Akaike information criterion (AIC) was selected. The multicollinearity between variables was checked using the variable inflation factor (VIF). The chi-square test and Pearson correlation were used with a significance threshold of 0.05. The association between underweight and oral lesions was evaluated using odds ratios (ORs) with 95% confidence intervals (CI).

## Results

### Sociodemographic characteristics of HIV positive and AIDS patients at Yaoundé Central Hospital, Cameroon

In our study, females were predominant, with a sex ratio of 1:2.5. The mean age was 43 (± 11) years, ranging from 18 to 73 years; 105 (51%) patients were aged between 30 and 59 years. Twenty-five (12%) had completed university, and 42 (20%) were unemployed (Table 1).

**Table 1:** Sociodemographic characteristics of HIV positive and AIDS patients at Yaoundé Central Hospital, Cameroon

Sociodemographic characteristics	*N* = 205^1^
Gender	
Female	147 (72)
Male	58 (28)
Age (in years)	
18–29	86 (42)
30–59	105 (51)
≥ 60	14 (6.8)
Level of education	
Primary school	49 (24)
Secondary school	131 (64)
University	25 (12)
Profession	
Employed	157 (77)
Student	6 (2.9)
Unemployed	42 (20)

^*1*^ N (%)

### Clinical and immunological characteristics of HIV positive and AIDS patients at Yaoundé Central Hospital, Cameroon

According to the WHO classification, 90 (44%) patients were in stage 3. Second line treatment was most common, with 38 patients (19%). The mean duration of treatment was 7.7 (± 2.4) months, ranging from zero to 21 months; 85 (41%) patients were receiving treatment between five and nine months. Considering the CD4 T-cell count, 21 (10%) patients had less than 200 cells/mm^3^ (Table 2).

**Table 2:** Clinical and immunological characteristics of HIV positive and AIDS patients at Yaoundé Central Hospital, Cameroon

Clinical and immunological characteristics	*N* = 205^1^	*P* value*
Clinical Characteristics		
WHO Clinical stage		**< 0.01**
Stage 1	40 (20)	
Stage 2	54 (26)	
Stage 3	90 (44)	
Stage 4	21 (10)	
Type of ARV treatment		**< 0.01**
First line	167 (81)	
Second line	38 (19)	
Duration of treatment (in years)		**< 0.01**
0–4	52 (25)	
5–9	85 (41)	
10–14	46 (22)	
≥ 15	22 (11)	
Immunological Characteristics		
CD4 T-cell count (Cellules/mm^3^)		**< 0.01**
< 200	21(10)	
200–500	154 (75)	
≥ 500	30 (15)	

^1^ n (%), first line (TDF/3TC/DTG), second line (ABC or AZT/3TC + ATV/r or LPV/r), *: Chi² test, bold: significant (*p* < 0.05)

### Oral health characteristics of HIV positive and AIDS patients at Yaoundé Central Hospital, Cameroon

The prevalence of oral lesions was 52.7% (95% CI: 45.6 to 59.6). The most common infection was candidiasis with 83 patients (40%), followed by gingival erythema and oral leukoplakia with 66 (32.2%) and 21 (10%), respectively. Oral hygiene assessment revealed that 121 patients (59%) had a thin layer of plaque, and 127 (62%) reported brushing their teeth once a day. The DMF index for our study population was 4.87 (Table 3).

**Table 3:** Oral health characteristics of HIV positive and AIDS patients at Yaoundé Central Hospital, Cameroon

Oral characteristics	*N* = 205^1^	*P* value*
Oral lesions		**0.044**
Candidiasis	83 (40.5)	
Gingival erythema	66 (32.2)	
Oral Leukoplakia	21 (10.2)	
Angular Cheilitis	17 (8.3)	
Ulceronecrotic periodontal disease	12 (6.9)	
Oral hygiene		**< 0.01**
No plaque	14 (6.8)	
Thin layer of plaque	121 (59)	
Visible plaque	57 (28)	
Plaque accumulation	13 (6.3)	
Frequency of brushing (number/day)		**< 0.01**
Once	127 (62)	
Twice	69 (34)	
Thrice	9 (4.4)	
State of teeth ²		**0.045**
DMF Index	4.8	
D Index	2.8	
M Index	1.4	
F Index	0.6	

^*1*^ n (%); ^*2*^ Mean, *: Chi² test, bold: significant (*p* < 0.05)

### Nutritional status of HIV positive and AIDS patients at Yaoundé Central Hospital, Cameroon

We found a mean weight of 67.5 (± 9.7) kg, ranging from 42.8 to 93 kg, while the mean height was 1.7 (± 0.08) m, ranging from 1.53 to 1.86 m. The mean body mass index was 23.35 (± 4.08) kg/m², ranging from 15.7 to 34.2 kg/m². Forty-one patients (20%) were underweight (95% CI: 14.88–26.26); 27 (13.2%) had mild (95% CI: 9-18.75), and 14 (6.8%) had moderate to severe (95% CI: 3.92–11.42%). Dietary analysis revealed that the food consumption score was poor to limited in 138 (67.3%) HIV positive patients (95% CI: 60.37–73.59). All these results are shown in Table 4.

**Table 4:** Nutritional status of HIV positive and AIDS patients at Yaoundé Central Hospital, Cameroon

Nutritional Status	*N* = 205^1^	*P* value
**Weight profile**		**< 0.01**
Underweight (yes)	41 (20)	
Light	27 (13.2)	
Moderate/severe	14 (6.8)	
**Food consumption score**		**< 0.01**
Poor/limited	138 (67.3)	
Acceptable	67 (32.7)	

^1^ n (%), *: Chi² test, bold: significant (*p* < 0.05)

### Association between oral pathologies and underweight in HIV positive and AIDS patients Yaoundé Central Hospital, Cameroon

Bivariate analysis revealed that the variables significantly associated with underweight were age (*p* = 0.002), WHO clinical stage (*p* = 0.001), duration of treatment (*p* < 0.001) and food consumption score (*p* < 0.001). Binary logistic regression revealed four models based on AIC values. The first model, which had an AIC of 167.8 and did not exclude any variables, was retained. Table 5 revealed that HIV positive and AIDS patients with oral pathologies were significantly 10.86 (95% CI: 2.48–16.63) more likely to be underweight than were HIV positive and AIDS patients without oral pathologies (*p* = 0.002).

**Table 5:** Association between underweight and oral pathologies in HIV positive and AIDS patients at Yaoundé Central Hospital, Cameroon

Characteristics	Underweight Yes, *N* = 98 ^1^ No, *N* = 107 ^1^		Crude Odd Ratio (IC À 95%) ^2^	Crude *P* value*	Adjusted Odd Ratio (IC À 95%) ^2^	Adjusted *P* value *
Oral lesions						
No	34 (35.1)	63 (64.9)	1	-	1	-
Yes	7 (6.5)	101 (93.5)	7.78 (3.2–18. 6)	< 0.01	10.86 (2.48–16.6)	0.002
Age (in years)						
>60	4 (28.6)	10 (71.4)	1	-	1	-
0–29	7 (8.1)	79 (91.9)	4.5 (1.1–18.1)	0.023	1.8 (0.3–10.9)	0.497
CD4 Count (cellules/mm^3^)						
≥ 500	3 (10)	27 (90)	1	-	1	-
200–500	35 (22.7)	119 (77.3)	0.38 (0.10–0.3)	0.014	0.086 (0.011–0.656)	0.018*
WHO Clinical staging						
Stage 4	3 (14.3)	18 (85.7)	1	-	1	-
Stage 2	21 (38.9)	33 (61.1)	0.26 (0.07–0.99)	0.054	1.04 (0.13–8.14)	0.97
Duration on ARV (in years)						
≥ 15	8 (36.4)	14 (63.6)	1	-	1	-
5–10	9 (10.6)	76 (89.4)	4.8 (1.6–14.6)	0.007	0.908 (0.70–4.84)	0.91
Food Consumption Score						
Poor/limited	38 (25.5)	100 (72.5)	8.1 (2.4-24.36)	-	1	-
Acceptable	3 (4.5)	64 (95.5)	0.469 (0.44–4.96)	< 0.01	2.37 (0.42–13.9)	0.329

^1^ n (%), ^2^ 95% CI: 95% confidence interval; *: Chi² (*n* > 5) and Pearson’s (*n* < 5) tests; bold: significant or close (*p* < 0.05)

## Discussion

The objective of this study was to assess the relationship between oral pathologies and underweight in HIV positive and AIDS patients at YCH in Cameroon.

In our study, 20% of the patients were at stage one. Our result is higher than that of Mindja Eko et al. in Cameroon in 2021, who reported 35.1% [6]. However, these findings are similar to those of studies conducted in West Africa, which reported a frequency between 40.6 and 45.33% [7. 8, 22]. This may be explained by the fact that the majority of patients are unaware of their status and generally discover their seropositivity at an advanced stage during routine examinations or by clinical suspicion following recurrent illnesses [6]. Our data corroborate this finding. Most of the HIV-positive and AIDS patients (41%) had been receiving treatment between five and nine months, which would suggest that their follow-up began at an advanced stage of the disease. Three out of four HIV-positive people had a CD4 count between 200 and 500 cells/mm³, while one in ten had a CD4 count of less than 200 cells/mm. Our results are similar to those reported by Minka Eko et al. in Cameroon in 2021: 53.6% of patients had a CD4 count between 200 and 500 cells/mm³, and 22.5% had a count less than 200 cells/mm³ [6]. However, these figures differ from those of Berberi et al. in Lebanon in 2015, who reported that 64% of patients had a c less than 200 cells/mm³ and 32% had a CD4 T-cell count between 200 and 500 cells/mm³ [23]. This situation could be the result of the stigmatization of HIV-positive patients, leading to a delay in access to care [7, 22].

The prevalence of oral pathologies was 47.3%. Higher proportions were reported previously in Mali (85.5%) and Cameroon (75.6%) (6,7). A low prevalence (31.4%) was also found in a West African study [24]. The most common oral lesion was candidiasis (40.5%). Several studies conducted in HIV positive and AIDS patients revealed that the most common lesion was candidiasis: 47.3% for Mindja Eko and al in Cameroon in 2021; 52.8% for Ba et al. in Mali in 2017 [6, 7]. However, higher prevalence of candidiasis were reported in Benin, Lebanon and Mali, 67%, 76% and 95%, respectively [8, 23, 24]. The predominance of this infection may be explained by the severe decline in immunity and the frequent use of antibiotics, both of which contribute to an imbalance in the biofilm of the oral cavity and favour its occurrence [8]. Gingival erythema was the second most common lesion, with a prevalence of 32.2%; this result is higher than that of a study carried out in 2017 in Mali, which reported 15.4%. Leukoplakia was diagnosed in 10.2% of patients. This prevalence is similar to that of a study carried out in Cameroon in 2021, which was 12.4% [6]. Gingival erythema and oral tongue leukoplakia are associated with a significant decrease in the CD4 count and are warning signs of progression to AIDS [8].

We found that oral hygiene was good in 59% of the HIV positive and AIDS patients, and 62% reported brushing their teeth once a day. Our results are similar to those of Mindja Eko et al. in Cameroon in 2021, who reported that 53.2% of HIV-positive patients had a thin layer of plaque, indicating good plaque control, and that 62.8% brushed their teeth once a day [6]. Another study revealed that 51.4% of the patients examined reported brushing their teeth at least twice a day [7]. The DMF index found in our study was 4.87. This result is greater than that of Mindja Eko et al. in Cameroon in 2021, who reported an index of 1.93 [6]. However, this value is still lower than that of Ba et al. in Mali in 2017, who reported an index of 10.17 [7]. Our study showed that few HIV-positive patients had received dental care. An explanation is that HIV positive and AIDS patients in Cameroon have limited financial resources to afford the high cost of dental care. Therefore, it is imperative to establish affordable dental care options for these patients [8].

The present study reported that the prevalence of underweight among HIV positive and AIDS patients at YCH was 20%. This result is higher than those reported by studies carried out in sub-Saharan Africa, which reported data between 5.8% and 13% [11, 12, 14, 25]. However, these findings are similar to those reported by Zemede et al. in Arba Minch, Ethiopia, in 2019 (18.2%) and Benzekri et al. in Senegal in 2015 (22.9%) [19, 26]. In addition, three other studies conducted in Ethiopia reported prevalences above 30%: Dehda et al. in Oromia in 2020 (30%), Daka et al. at Jimma Medical Centre in 2020 (34%) and Habtamu et al. at the Jimma Specialized University Hospital in 2018 (46.8%) [13, 24, 27]. The differences in the prevalence of underweight individuals are due to socioeconomic disparities and variations in eating habits [13, 24, 25, 32]. Nutrition plays an important role in immunity and influences the immune system’s ability to respond to infections [27]. In our study, 67.3% of the HIV positive and AIDS patients had a poor to limited food consumption score, meaning that they did not meet nutritional requirements in terms of food quantity and quality. Diet is a key element in the management of people infected with HIV [28–31]. Studies have also shown that underweight is one of the major signs of advanced-stage patients [32, 33]. Our study results of mild (13.2%) and 6.8% moderate-to-severe (6.8%) forms of underweight were comparable to those of studies reporting 12.7 to 25% mild and 5.6 to 9% moderate [13, 16, 32, 34].

We found that HIV-positive and AIDS patients with oral pathologies were significantly 10.86 times (95% CI: 2.48–16.63) more likely to be underweight than were those without oral pathologies. The development of oral pathologies depends on the interaction between the host response and the oral microbial biofilm, which comprises many microorganisms [35]. Many factors can influence the development of these lesions, including advanced age, smoking habits, failure to comply with oral hygiene rules and an impaired immune response [35]. Additionally, HIV infection leads to impaired immunity, resulting in the appearance of opportunistic infections, including lesions in soft tissues of the oral cavity [13, 34, 36]. These oral pathologies, associated with pain, lead to reduced food intake and, consequently, an inadequate diet. In the long run, this will result in undernutrition and underweight [37–40].

## Conclusion

In our study, a quarter of the HIV-positive and AIDS patients were underweight. Oral examination revealed that more than half of the patients had oral pathologies, with oral candidiasis, gingival linear erythema and oral leukoplakia being the most common. Additionally, HIV-positive and AIDS patients were at high risk of being underweight. The effective medical care of these patients must include oral and nutritional management to improve their health and quality of life.

### Electronic supplementary material

Below is the link to the electronic supplementary material.

Supplementary Material 1

## Data Availability

The data from our study are available upon reasonable request from the corresponding author of the manuscript.

## Abbreviations

ABC: Abacavir
AIC: Akaike information criterion
ATV/r: Atazanavir enhanced with ritonavir
AZT: Zidovudine
AIDS: Acquired immune deficiency syndrome
BMI: Body mass index
CSPro: Census and Survey Processing System
CDC: Center for Disease Control and Prevention
DMF: Decayed missed and filled tooth
DTG: Dolutegravir
FCS: Food consumption score
HIV: Human immunodeficiency virus
LPV/r: Lopinavir boosted with Ritonavir
UNAIDS: United Nations AIDS Program
VIF: Variable Inflation Factor
WFP: World Food Program
WHO: World Health Organization
YCH: Yaoundé Central Hospital
TDF: Ténofovir
3TC: Lamivudine
