# Supplementary material for: Oral pathologies and underweight conditions among people living with HIV/AIDS in a health facility in Yaoundé, Cameroon: a study of 205 cases
Source: BMC Nutr. 2024 Feb 15;10:30. doi: 10.1186/s40795-024-00835-z (PMC10867986; doi:10.1186/s40795-024-00835-z)
Supplement: Supplementary file 1 — Supplementary Material 1 [file 40795_2024_835_MOESM1_ESM.docx]

**QUESTIONNAIRE**

N° : Date: / /

## Section one: Sociodemographic characteristics

## 1. Sex: | | 1) Male; 2) Female

## 2. Age (in years): …………….

## 3. Profession: | | 1) Pupil/student 2) Employed; 3) Unemployed

## 4. Level of education: | | 1) Primary; 2) Secondary; 3) University

## Section two: history of HIV

## 5. Date of discovery of HIV (dd/mm/yy) :........................................

## 6. Treatment initiation date (dd/mm/yy):.................................

## 7. ARV treatment: | | 1) Yes; 2) No

## 8. If yes, specify the ARV line (in months): | | 1) First (tenofovir, TDF/lamivudine, 3TC/dolutegravir, DTG); 2) Second line (abacavir, ABC or zidovudine, AZT/lamivudine, 3TC + atazanavir boosted with ritonavir, ATV/r or lopinavir boosted with ritonavir, LPV/r); 3) Third (dugunavir boosted with ritonavir, DRV/r) + dolutegravir (DTG) ± 1-2 nucleotide reverse transcriptase inhibitors)

## 9. CD4+ T-cell count (last six months in cells/mm^3^):........................

## 10. Specify the patient's clinical stage according to the WHO: | | 1) Stage 1, 2) Stage 2, 3) Stage 3, and 4) Stage 4.

## Section three: Oral health assessment

## 11. Oral hygiene: | | 1) no plaque, 2) thin layer, 3) visible plaque deposit, and 4) plaque accumulation

## 12. Specify the condition of each tooth (Decayed Missed Filled) after examination:

| Tooth number | | | 48 | | 47 | | 46 | | 45 | | 44 | | 43 | | 42 | | 41 | | 31 | | 32 | | 33 | | 34 | | 35 | | 36 | | 37 | | 38 |
| --- | --- | --- | --- | --- | --- | --- | --- | --- | --- | --- | --- | --- | --- | --- | --- | --- | --- | --- | --- | --- | --- | --- | --- | --- | --- | --- | --- | --- | --- | --- | --- | --- | --- |
| D | | |  | |  | |  | |  | |  | |  | |  | |  | |  | |  | |  | |  | |  | |  | |  | |  |
| M | | |  | |  | |  | |  | |  | |  | |  | |  | |  | |  | |  | |  | |  | |  | |  | |  |
| F | | |  | |  | |  | |  | |  | |  | |  | |  | |  | |  | |  | |  | |  | |  | |  | |  |
|  | |  |  | |  | |  | |  | |  | |  | |  | |  | |  | |  | |  | |  | |  | |  | |  | |  |

| Tooth number | 18 | 17 | 16 | 15 | 14 | 13 | 12 | 11 | 21 | 22 | 23 | 24 | 25 | 26 | 27 | 28 |
| --- | --- | --- | --- | --- | --- | --- | --- | --- | --- | --- | --- | --- | --- | --- | --- | --- |
| D |  |  |  |  |  |  |  |  |  |  |  |  |  |  |  |  |
| M |  |  |  |  |  |  |  |  |  |  |  |  |  |  |  |  |
| F |  |  |  |  |  |  |  |  |  |  |  |  |  |  |  |  |

## 13. 13. Report the pathologies found :

## Oral candidiasis: | | 1) Yes 2) No

## Oral hairy leukoplakia: | | 1) Yes 2) No

## Kaposi sarcoma: | | 1) Yes 2) No

## Ulceronecrotic periodontitis: | | 1) Yes 2) No

## Ulceronecrotic gingivitis: | | 1) Yes 2) No

## Linear gingival erythema: | | 1) Yes 2) No

## Herpetic lesions: | | 1) Yes 2) No

## Zona: | | 1) Yes 2) No

## Mouth ulcers: | | 1) Yes 2) No

## Other pathology:

## Section four: Anthropometric features

## 14. Weight:...................(kg)

## 15. Height:.....................(m)

## 16. Waist size:.............(cm)

## 17. Skin fold: | | 1) Elastic; 2) Returns slowly (< 2 secondes); 3) Returns very slowly (> 2 secondes)

## Fifth section: Dietary habits assessed with the 24-hour recall method

## 18. Specify the frequency of consumption of food products and/or dietary supplements made from cereals/starches (corn, e.g., corn-fu, 'corn-chaff,'pop-corn, etc., wholemeal bread, white bread, white rice, wholemeal rice, millet, pasta e.g., spaghetti, macaroni, wheat products, e.g., bread, pizza, cornflakes, "puff pastry", plantain, manioc, yam and its derivatives, sweet potato, potato )? | | 1) Once 2) Twice 3) Three times 4) Four times 5) Five times 6) Six times 7) Seven times or more

## 19. Specify the frequency of consumption of food products and/or dietary supplements made from pulses (corn, e.g., beans, nuts)? | | 1) Once 2) Twice 3) Three times 4) Four times 5) Five times 6) Six times 7) Seven times or more

## 20. Specify the frequency of consumption of food products and/or dietary supplements made from meat products (fish, pork, red meat, poultry, e.g., chicken, duck, sardines, sausage, core, and eggs) was calculated as follows: | | 1) Once 2) Twice 3) Three times 4) Four times 5) Five times 6) Six times 7) Seven times and more

## 21. Specify the frequency of consumption of food products and/or food supplements made from vegetables (beans, peanuts, cows, soybeans, green leaves) was calculated as follows: | | 1) Once 2) Twice 3) Three times 4) Four times 5) Five times 6) Six times 7) Seven times or more

## 22. Specify the frequency of consumption of food products and/or dietary supplements made from milk and dairy products (skim milk, whole milk, yogurt, and hard cheese) was calculated as follows: | | 1) Once 2) Two times 3) Three times 4) Four times 5) Five times 6) Six times 7) Seven times or more

## 23. Specify the frequency of consumption of food products and/or dietary supplements made from fruits (corossole, banana, pineapple, Citrus, grapefruit, apple, avocado, watermelon, papaya, mango, and guava): | | 1) Once 2) Twice 3) Three times 4) Four times 5) Five times 6) Six times 7) Seven times or more

## 24. Specify the frequency of consumption of food products and/or dietary supplements made with sugars/fatty foods (honey, table or refined sugar, sweetener) : | | 1) Once 2) Twice 3) Three times 4) Four times 5) Five times 6) Six times 7) Seven times or more

## 25. Specify the frequency of consumption of food products and/or dietary supplements made from fats (e.g., refined oil, Mayor, Azur, star oil, Jadida, Diamaor, and soybean oil, e.g., soyor, Aya oil and sesame oil): | | 1) Once 2) Twice 3) three times 4) four times 5) five times 6) six times 7) seven or more
